# Evidence of Prolonged Crimean-Congo Hemorrhagic Fever Virus Endemicity by Retrospective Serosurvey, Eastern Spain

**DOI:** 10.3201/eid2805.212335

**Published:** 2022-05

**Authors:** Laura Carrera-Faja, Jesús Cardells, Lola Pailler-García, Víctor Lizana, Gemma Alfaro-Deval, Johan Espunyes, Sebastian Napp, Oscar Cabezón

**Affiliations:** Universitat Autònoma de Barcelona, Bellaterra, Spain (L. Carrera-Faja, J. Espunyes, O. Cabezón);; Universidad Cardenal Herrera-CEU, Valencia, Spain (J. Cardells, V. Lizana, G. Alfaro-Deval);; Institut de Recerca i Tecnologia Agroalimentàries, Bellaterra (L. Pailler-García, S. Napp)

**Keywords:** Crimean-Congo hemorrhagic fever, viruses, zoonoses, parasites, tickborne disease, vector-borne infections, emerging infections, Ixodidae, *Hyalomma* ticks, Mediterranean, ungulates, Spain

## Abstract

We conducted a retrospective serosurvey for antibodies against Crimean-Congo hemorrhagic fever virus in wild ungulates along the eastern Mediterranean Coast of Spain. The virus has been endemic in this region since 2010 but is mainly restricted to geographic clusters with extremely high seropositivity associated with high density of bovids.

Crimean-Congo hemorrhagic fever (CCHF) is caused by CCHF virus (CCHFV), a tickborne pathogen of the genus *Orthonairovirus*, belonging to the family *Bunyaviridae*. In humans, CCHFV can induce a severe and potentially fatal systemic hemorrhagic disease. CCHFV infections in wildlife and domestic animals are generally subclinical but, in some species, can induce enough viremia to enable virus transmission to uninfected ticks. Moreover, infected animals produce antibodies, enabling the identification of affected areas through retrospective serologic studies (*1*). CCHFV is endemic in several countries in Asia, Africa, the Middle East, and southeastern Europe and has a range similar to that of its main vectors and reservoirs, *Hyalomma* spp. ticks, which are expanding their habitat range in southern Europe (*2*).

In Spain, CCHFV was detected in *H. lusitanicum* ticks from a red deer (*Cervus elaphus*) in 2010 (*3*). Since 2013, several severe CCHF cases in humans have been reported in the country (*4*). Viral strains identified in Spain showed high genetic variability, suggesting repeated introductions from different origins, including Africa and eastern Europe (*5*,*6*). Seroprevalence studies conducted in 2017 and 2018 showed evidence that CCHFV is prevalent over large areas of central and southern Spain, which coincide with the regions where *H. marginatum* and *H. lusitanicum* ticks have been described (*4*,*6*). Along the Mediterranean Coast of eastern Spain, the existence of CCHFV vectors (*Hyalomma* ticks) and of the virus itself were uncertain until recently, when *H. lusitanicum* ticks were found in wild boars (*Sus scrofa*) from the metropolitan area of Barcelona (*7*), and CCHFV seropositivity was reported in ungulates from southern Catalonia (*8*). To evaluate the extent and duration of CCHFV circulation in eastern Spain, we conducted a retrospective serosurvey to detect CCHFV antibodies in different wildlife species in the Valencia region.

## The Study

We used the CCHF Double Antigen Multi-Species ELISA kit (IDvet, https://www.id-vet.com) to test for CCHFV antibodies in serum samples collected from 332 wild boars, 126 Iberian ibexes (*Capra pyrenaica*), and 48 mouflons (*Ovis aries musimon*). Serum samples were collected during 2010–2021 within the framework of the wildlife surveillance program in the Valencia region. We chose wild boars, Iberian ibexes, and mouflons because they are the main wild ungulate species in the region. Iberian ibexes and mouflons were selected from the 2 areas where they are more abundant. We also selected serum samples taken from boars in the same 2 areas and from areas with low densities of wild ruminants. (Appendix Figure 1).

Our results showed that CCHFV was already circulating in different areas of the Valencia region by the time the virus was reported in Spain in 2010 (Table; Appendix Figure 2). These results are consistent with the phylogenetic analysis of the CCHFV strain obtained from a *H. lusitanicum* tick collected in western Spain in 2014 that suggested the strain had been circulating in the country for several decades (*9*). Together with the variability of CCHFV strains identified in Spain (*5*,*6*), our findings suggest an epidemiologic scenario in which CCHFV has been repeatedly introduced into different regions of Spain over many years.

**Table:** Seropositivity of serum samples from various mammalian species tested for antibodies against Crimean-Congo hemorrhagic fever virus, Valencia region, Spain*

Year	Iberian ibex (*Capra pyrenaica*)	Mouflon (*Ovis aries musimon*)	Wild boar (*Sus scrofa*)	Total
2010	–	–	21/84 (17–36)	21/84 (17–36)
2011	–	–	12/92 (7–22)	12/92 (7–22)
2012	–	–	4/50 (3–20)	4/50 (3–20)
2013	–	–	0/12 (0–30)	0/12 (0–30)
2014	–	–	8/40 (10–36)	8/40 (10–36)
2015	–	–	6/49 (6–26)	6/49 (6–26)
2016	–	–	0/4 (0–60)	0/4 (0–60)
2017	13/13 (72–100)	–	–	13/13 (72–100)
2018	38/39 (85–100)	15/15 (75–100)	0/1 (0–95)	53/55 (86–99)
2019	51/54 (84–99)	33/33 (87–100)	–	84/87 (90–99)
2020	16/17 (69–100)	–	–	16/17 (69–100)
2021	3/3 (31–100)	–	–	3/3 (31–100)
Total	121/126 (91–99)	48/48 (91–100)	51/332 (12–20)	220/506 (39–48)

*Data are no. positive/no. tested (95% CI for percent seropositive). –, no samples tested.

Among Iberian ibex serum samples from Valencia, 96.0% (121/126) had antibodies against CCHFV, which is close to the 100% seroprevalence reported for the same species in the affected neighboring area of Catalonia (*8*). Likewise, all the mouflon (48/48) samples in this study were seropositive, indicating a high susceptibility in this species, even though CCHFV infection has not been previously described in mouflons. In contrast, only 15.5% (51/332) of the wild boar samples tested were seropositive, and wild boars in the areas of high densities of Iberian ibexes and mouflons had seroprevalences of only 36.0% (49/136), which coincides with the results obtained in Catalonia (*8*). One possible explanation for the prevalences we found in Iberian ibexes, mouflons, and wild boars is that *Hyalomma* genus ticks feed preferably on species of the family *Bovidae* but also feed, although less prominently, in the family *Suidae* (*10*).

CCHFV seropositivity in the Valencia region clustered in 2 areas (Figure, panel A). One cluster was in the north in the Tinença de Benifassà Natural Park, an area of the region that is a continuation of the Ports de Tortosa-Beseit National Game Reserve, the affected area in Catalonia that is close to the Ebro Delta wetland (*8*). The other cluster was located at the Muela de Cortes y el Caroche natural area in central Valencia region, <40 km from the Albufera, the third-largest wetland in Spain. Identifying 2 main CCHFV transmission areas close to key stopover areas for migratory birds adds weight to the hypothesis of CCHFV introduction in Spain via migratory birds carrying infected ticks. In fact, the Mediterranean/Black Sea Flyway and the East Atlantic Flyway, 2 of the 3 Palaearctic-African flyways connecting Europe with Africa, converge on the Mediterranean Coast of eastern Spain. 

**Figure:**
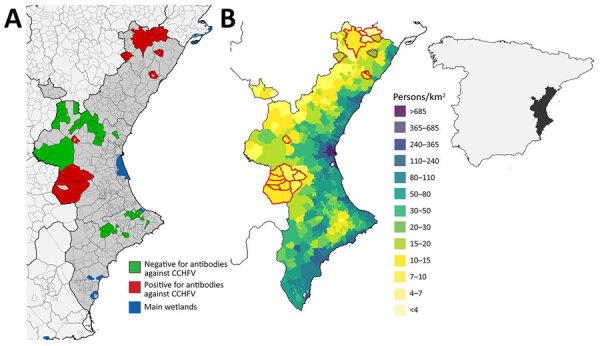
Crimean-Congo hemorrhagic fever virus (CCHFV) seropositivity in Iberian ibexes (*Capra pyrenaica*), mouflons (*Ovis aries musimon*), and wild boars (*Sus scrofa*), Valencia region, Spain, 2010–2021. A) Areas in Valencia where tested animals were seropositive and seronegative. Green indicates all samples were seronegative; red indicates >1 sample was seropositive; gray indicates areas not sampled. Asterisk (*) indicates Chera and dagger (†) indicates Vilanova d'Alcolea, 2 areas of CCHFV-seropositivity in wild boars outside the main areas in which Iberian ibexes and mouflons tested positive. B) Density of human population, Valencia region, Spain 2015. Areas with red outlines coincide with areas in which CCHFV-seropositive animals were sampled. Map at right shows the Valencia region in Spain.

We also detected CCHFV antibodies in a few wild boars outside the 2 main positive areas (Figure, panel A). Because wild boars are known to disperse over long distances (*11*), this species could play a key role in the spread of CCHFV outside endemic areas.

A recent study mapped the risk for CCHFV exposure among humans in mainland Spain by using red deer as an indicator of the transmission risk plus environmental variables (*12*), but that study did not predict areas of high risk that we identified in the Valencia region or those identified farther north (*8*). Those findings indicate that determinants of CCHFV circulation in central and southwestern Spain are clearly different from those in the Mediterranean area, where Iberian ibexes, and to a lesser extent wild boars and mouflons, likely play a key role.

Little information is available on the distribution of competent CCHFV vectors in the Valencia region, but a study to the north of the region reported a substantial increase during 2017–2018 in the number of persons receiving tick bites, 85% of which were caused by *H. lusitanicum *ticks (*13*). Other studies have suggested that the lack of human CCHF cases in the Mediterranean region, despite areas with widespread CCHFV, is the result of a low rate of contact between humans and infected ticks (*14*). At least in the Valencia region, this low contact seems to be the case; areas where CCHFV transmission in wildlife is concentrated coincide with the areas with the lowest human density (Figure, panel B). However, rising wild ungulate populations that are moving closer to densely populated areas could change the human epidemiologic situation.

## Conclusions

Our results support an epidemiologic scenario in which CCHFV has been endemic in wild ungulates in different regions of Spain before it was detected in 2010. In eastern Spain, CCHFV circulation mainly occurs in geographic clusters associated with high densities of *Bovidae* species. However, as these species move into areas with higher human populations, more human CCHF cases could occur. To protect the population of the region, public health authorities should continue CCHFV surveillance among tick and ungulate species.

Appendix Additional information on prolonged Crimean-Congo hemorrhagic fever virus endemicity detected by retrospective serosurvey, eastern Spain.
