# Supplementary material for: Evidence of Prolonged Crimean-Congo Hemorrhagic Fever Virus Endemicity by Retrospective Serosurvey, Eastern Spain
Source: Emerg Infect Dis. 2022 May;28(5):1031–4. doi: 10.3201/eid2805.212335 (PMC9045418; doi:10.3201/eid2805.212335)
Supplement: Appendix — Additional information on prolonged Crimean-Congo hemorrhagic fever virus endemicity detected by retrospective serosurvey, eastern Spain. [file 21-2335-Techapp-s1.pdf]

Article DOI: https://doi.org/10.3201/eid2805.212335

Evidence of Prolonged Crimean-Congo Hemorrhagic Fever Virus Endemicity by Retrospective Serosurvey, Eastern Spain

Appendix

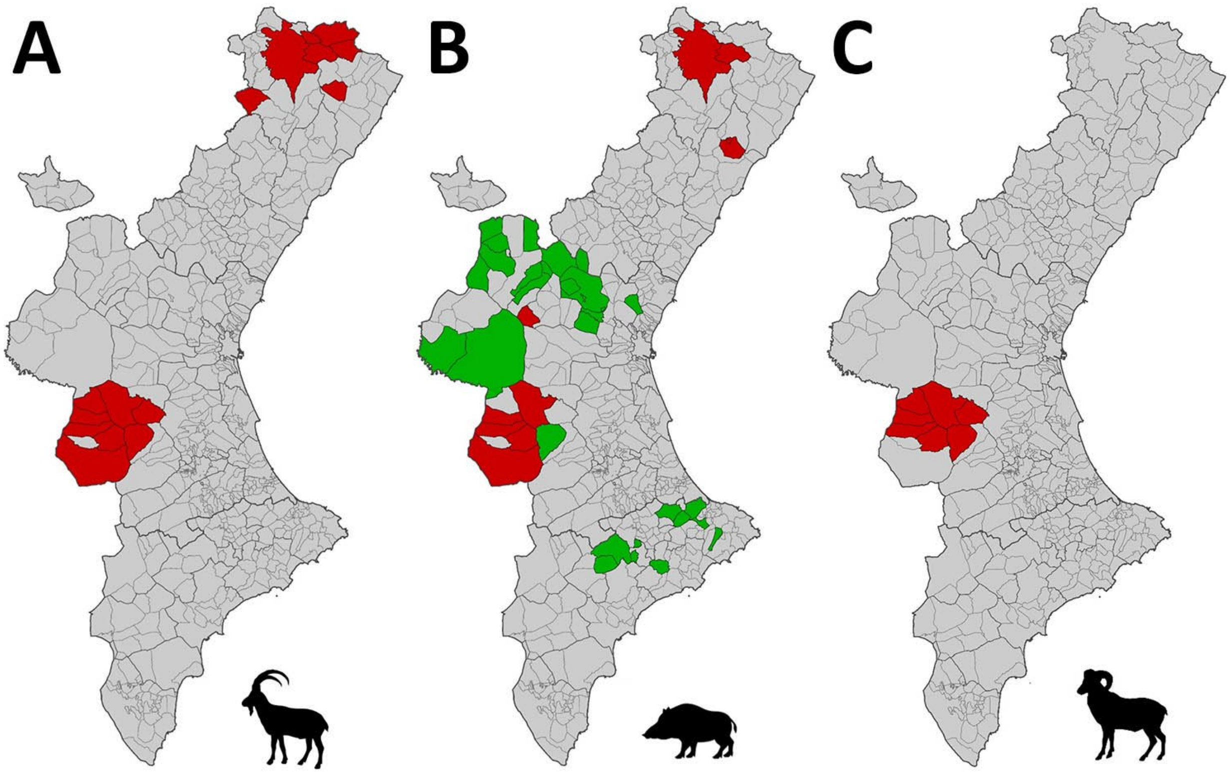

Appendix Figure 1. Distribution of areas sampled for detection of antibodies against Crimean-Congo hemorrhagic fever virus (CCHFV) by species, Valencia region, Spain. A) Iberian ibex (*Capra pyrenaica*); B) wild boar (*Sus scrofa*); C) European mouflon (*Ovis aries musimon*). Red indicates areas in which $\geq$1 animals tested CCHFV seropositive; green indicates areas in which all animals tested seronegative.

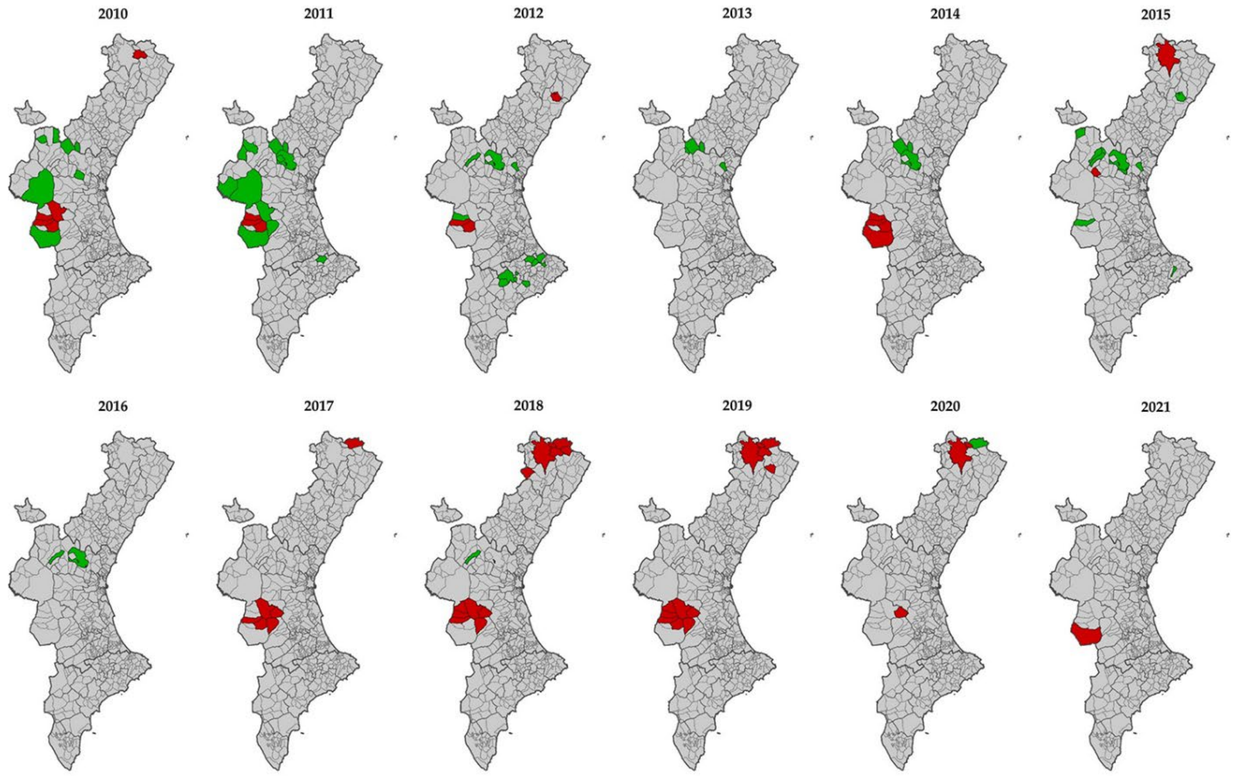

Appendix Figure 2. Annual Crimean-Congo hemorrhagic fever virus seropositivity in wild boars (*Sus scrofa*), Iberian ibexes (*Capra pyrenaica*), and mouflons (*Ovis aries musimon*), Valencia region, Spain, 2010–2021. Red indicates areas in which $\geq$1 animals tested seropositive; green indicates areas in which all animals tested seronegative.
